# Elevated HERV-K Expression in Soft Tissue Sarcoma Is Associated with Worsened Relapse-Free Survival

**DOI:** 10.3389/fmicb.2018.00211

**Published:** 2018-02-13

**Authors:** Maria Giebler, Martin S. Staege, Sindy Blauschmidt, Lea I. Ohm, Matthias Kraus, Peter Würl, Helge Taubert, Thomas Greither

**Affiliations:** ^1^Center for Reproductive Medicine and Andrology, Martin Luther University of Halle-Wittenberg, Halle, Germany; ^2^Department of Pediatrics I, Martin Luther University of Halle-Wittenberg, Halle, Germany; ^3^Department of General, Visceral and Thoracic Surgery, Städtische Klinikum Dessau, Dessau-Roßlau, Germany; ^4^Division Molecular Urology, Department of Urology and Pediatric Urology, University Hospital Erlangen, Friedrich-Alexander-Universität Erlangen-Nürnberg, Erlangen, Germany

**Keywords:** soft tissue sarcoma, HERV-K, HERV-Fb, prognosis, relapse

## Abstract

A wide variety of endogenous retroviral sequences has been demonstrated in the human genome so far, divided into several different families according to the sequence homology to viral strains. While increased expression of human endogenous retrovirus (HERV) elements has already been linked to unfavorable prognosis in hepatocellular carcinoma, breast cancer, and ovarian carcinoma yet less is known about the impact of the expression of different HERV elements on sarcomagenesis in general as well as the outcome of soft tissue sarcoma (STS) patients. Therefore, in this study the association between expression of HERV-K and HERV-F and the clinicopathological characteristics in a cohort of STSs as well as the patients’ prognosis was evaluated. HERV-K and HERV-F expression was assessed by quantitative real-time PCR in 120 patient specimens. HERV-K and HERV-F expression was significantly correlated (*r*_S_ = 0.5; *p* = 6.4 × 10^-9^; Spearman’s rank bivariate correlation). Also, tumor diameter exhibited a significant negative association to HERV-K and HERV-F expression. Levels of several hypoxia-related RNAs like HIF-1α and miR-210 showed a significant positive correlation with both HERV-K and HERV-F expression. Although in survival analyses no impact of HERV expression on disease-specific survival could be detected, patients with elevated HERV-K expression had a significantly shorter relapse-free survival (*p* = 0.014, log-rank analysis). In conclusion, we provide evidence for the first time that the increased expression of HERV-K in tumors is associated with STS patients’ prognosis.

## Introduction

Soft tissue sarcomas (STSs) are a heterogeneous group of tumors classified by the somatic tissue they resemble, with over 50 subtypes that can be distinguished (Ducimetière et al., 2011). The incidence of STS is relatively low – with estimated 4–5 cases per 100,000 per year (Stiller et al., 2013) – but the 5-year survival is only around 50% (Ferrari et al., 2011). Although treatment of STS usually consists of a wide resection of the tumor, followed by radio- or chemotherapy in selected cases (Casali and Blay, 2010), relapses and metastases are still an urgent clinical issue (Steinestel and Wardelmann, 2015). Due to the heterogeneity in genetics and phenotypes of the different STS entities, many of the proposed laboratory biomarkers and clinical factors still are insufficient for a satisfying prognostic evaluation of the individual patient’s outcome.

Human endogenous retroviruses (HERVs) are a class of retroviral sequences acquired during evolution by integration of viral genes in the host genome. They became non-infectious by mutation or loss of relevant genes for replication or virus release (Vargiu et al., 2016). They comprise an estimated 8% of the human genome (Lander et al., 2001), and at least 22 independent families based on their homology to known mammalian retroviruses exist (Griffiths, 2001).

Among the different families, retroviral sequences of the HERV-K family (HML-2) were the latest acquired, therefore they are the most complete and biologically active family (Bannert and Kurth, 2004; Hughes and Coffin, 2004, 2005). HERV-K expression was detected in several tumor entities, among them chronic myeloid leukemia (Brodsky et al., 1993), renal cell carcinoma (Florl et al., 1999; Kreimer et al., 2013), breast carcinoma (Wang-Johanning et al., 2003), prostate carcinoma (Wallace et al., 2014), pancreatic cancer (Li et al., 2017), and melanoma (Serafino et al., 2009). In melanoma, HERV-K expression is suggested to be an early event in tumorigenesis, seemingly enhancing the pathological process of tumor formation (Serafino et al., 2009; Cegolon et al., 2013). Most recently, it was demonstrated that shRNA-mediated downregulation of HERV-K in pancreatic cancer cell lines suppressed growth rates and metastases as well as the expression of several proliferation-related genes (Li et al., 2017). However, it is worth mentioning that the above described associations between HERV expression and tumor tissues are correlative. It has also been demonstrated, that healthy tissues of different origins express HERV sequences, especially during early embryo development and placentation or in the innate immune response (reviewed in Meyer et al., 2017).

HERV-F is another family of HERVs originally identified by cloning of human genomic DNA sequences and homology analyses (Kjellman et al., 1999a,b). HERV-F expression was described in leukemia cell lines (Patzke et al., 2002) and in a wide range of other tumor cell lines (Yi and Kim, 2004). On the contrary, in adult somatic tissues HERV-F is only expressed in placenta (Kjellman et al., 1999a; Yi and Kim, 2004). Recently, constitutive HERV-F expression was reported in a cohort of breast cancer patients, with an increased expression of HERV-F members in comparison to normal breast tissue (Frank et al., 2008).

The knowledge on the expression and clinical impact of HERV-K and HERV-F family in STS is scarce. Schiavetti et al. (2002) reported a robust expression of HERV-K-MEL in human sarcoma specimens, which was higher than in other tumor entities and comparable to the expression in bladder and breast carcinoma, but lower than in melanoma samples. We hypothesized that a detectable HERV mRNA expression could be a common feature in STS and might be related to the patients’ clinical outcome. Therefore, the aim of this study was the quantification of HERV-K and HERV-F family mRNA expression in a cohort of 120 STS samples and the correlation to clinicopathological and prognostic data of the patients. Furthermore, as a secondary end point we analyzed the correlation of the mRNA expression of HERV-K and HERV-F with the RNA expression of known apoptosis-related [B-cell cll/lymphoma 2 (BCL2)] or hypoxia-related (miR-210, miR-199a, Hypoxia inducible factor 1a) genes as well as known epigenetically regulated genes (miR-203, H2A.Bbd).

## Materials and Methods

### Patients

One hundred and twenty STS patients agreed to participate in this study. An overview of the patient cohort is given in **Table 1**. Patients underwent tumor surgical resection between 1998 and 2001 at the Department of Surgery, University of Leipzig (Leipzig, Germany) without prior adjuvant treatment. Thirty-nine patients exhibited metastases (32.5%). Fresh tumor tissue was snap-frozen immediately after excision and stored at -80°C until RNA isolation. The study was approved by the local ethics committee of the Medical Faculty of the Martin Luther University of Halle-Wittenberg and the Medical Faculty of the University of Leipzig. According to the Helsinki Declaration, all patients gave written informed consent. Patient cohort composition as well as tissue cryopreservation was as described previously (Kappler et al., 2001; Würl et al., 2002).

**Table 1:** Clinical and histopathological characteristics in relation to HERV-K and HERV-F mRNA expression.

		HERV-K low (<0.56)	HERV-K high (>0.56)	Chi^2^ test (*p*-value)	HERV-F low (<2.02)	HERV-F high (>2.02)	Chi^2^ test (*p*-value)
Age	<60 years	28	35	n.s.	31	32	n.s.
	>60 years	32	25		29	28	
Sex	Female	25	30	n.s.	25	30	n.s.
	Male	35	30		35	30	
Patients status	Alive	34	27	n.s.	31	30	n.s.
	Deceased	26	33		29	30	
Tumor stage^a^	I	9	4	n.s.	7	6	n.s.
	II	21	34		25	30	
	III	22	17		21	18	
	IV	8	5		7	6	
Resection	Radical (R0)	41	43	n.s.	39	45	n.s.
	Not radical (R1)	19	17		21	15	
Tumor localization	Extremities	34	42	n.s.	40	36	n.s.
	Trunk wall	6	4		4	6	
	Head/neck	2	2		3	1	
	Abdomen/peritoneum	17	9		12	14	
	Multiple locations	1	1		1	1	
Histological subtypes	LS	15	9	n.s.	15	9	**0.047**
	FS	2	4		1	5	
	RMS	5	3		5	3	
	LMS	12	7		6	13	
	NS	4	10		4	10	
	Syn	2	8		4	6	
	NOS	15	16		19	12	
	Other	5	3		6	2	
Tumor size	T1	9	10	n.s.	7	12	n.s.
	T2	51	50		53	48	
Number of relapses	0	43	31	(0.077)	37	37	n.s.
	1	7	13		10	10	
	>2	10	16		13	13	
Metastases	M0	45	36	n.s.	46	35	n.s.
	M1	15	24		14	25	


*One significant association (Chi^*2*^ test) is marked in bold. ^*a*^Union for International Cancer Control Guidelines; LS, liposarcoma; FS, fibrosarcoma; RMS, rhabdomyosarcoma; LMS, leiomyosarcoma; NS, neuronal sarcoma; Syn, synovial sarcoma; NOS, not other specified; T1, <5 cm in diameter; T2, >5 cm in diameter.*

### RNA Isolation

Tissue specimens were partly processed on a cryotome in 5 μm tissue slices, and RNA was isolated from 20 tissue slices. The slices were incubated in Trizol (Thermo Fisher Scientific, Waltham, MA, United States) for 5 min at room temperature and subsequently mixed with chloroform (AppliChem, Darmstadt, Germany). After centrifugation, aqueous phase was collected and treated with DNase (Qiagen, Hilden, Germany). Total RNA was precipitated with isopropanol (AppliChem, Darmstadt, Germany) for 12 h at 4°C, washed with different ice-cooled ethanol solutions (96 and 70%) and finally dissolved in RNase-free water (Qiagen, Hilden, Germany). RNA concentrations were assessed spectrometrically.

### cDNA Synthesis and qPCR

cDNA synthesis was carried out with RevertAid First strand synthesis kit (Thermo Fisher Scientific, Waltham, MA, United States) according to manufacturer’s protocol. One μg total RNA was applied for cDNA synthesis per tissue specimen. The complete elimination of genomic DNA was controlled by mock-RT PCR reactions (see Supplementary Figures S1, S2). cDNA was quantified with Maxima SyBR Green Kit (Thermo Fisher Scientific, Waltham, MA, United States) in a quantitative real-time-PCR reaction. The applied primer sequences for the PCR reaction were: HERV-K forward: 5′-GGC CAT CAG AGT CTA AAC CAC G-3′; HERV-K reverse: 5′-CTG ACT TTC TGG GGG TGG CCG-3′; HERV-F forward: 5′-CCT CCA GTC ACA ACA ACT C-3′; HERV-F reverse: 5′-TAT TGA AGA AGG CGG CTG G-3′ (Seifarth et al., 2005); H2A.Bbd forward: 5′-TCG TTT TCA GTA GCC AGG T-3′; H2A.Bbd reverse: 5′-CAG AAT TAA TGA AGG CCC AAG-3′; HPRT forward: 5′-TTG CTG ACC TGC TGG ATT AC-3′; HPRT reverse: 5′-CTT GCG ACC TTG ACC ATC TT-3′. Samples were run on a MyIQ cycler (BioRad, Hercules, CA, United States) and HERV-K or HERV-F expression calculated according to the 2^-ΔCT^ method (Schmittgen and Livak, 2008) with HPRT as reference gene. Linearity of the qPCR reaction for both HERV-K and HERV-F was analyzed by dilution series of the gel-extracted amplicon (see Supplementary Figure S3). All amplicons were analyzed by qPCR melt analyses on the occurrence of a single, distinct peak (see Supplementary Figures S4, S5). Representative PCR products were purified by agarose gel electrophoresis and subsequently sequenced (see **Table 2**). Analysis of the sequenced PCR products with RepeatMasker ^1^ demonstrated that the used primers amplified sequences from HERV-K and HERV-Fb (HERVFH21). Expression analyses for BCL2 mRNA, miR-203 and miR-210 (Greither et al., 2012), HIF-1α mRNA (Kessler et al., 2010) and miR-199a (Keßler et al., 2016) were carried out as previously described.

**Table 2:** Sequence information on HERV-K and HERV-F amplicons.

	Amplicon length (bp)	qPCR melt analysis	Confirmed by sequencing	Amplicon sequence
HERV-K	167	Single peak	Yes	5′-GGCCATCAGAGTCTAAACCACGAGGNACAAGT CCTCTTCCAGCAGGTCAGGT GCCNGTAACATTACAACCTCAAAcGCAGGTTAAAGAAAATAAGACCCAA CCGCC AGTAGCYTATCAATACTGGCCGCCGGCTGAACTTCAGTATCGGCCACCCCC AGAAAGTCAG-3′
HERV-F	129	Single peak	Yes	5′-CCTCCAGTCACAACAACTCACGTGG ACTGTCCTCCCTCAGGNCTTCCAG GATAGCCttcttttctTCGGGCAAGCCCTAGCCCAAGACCTTGCCTCCTTGGATCTTT CCCCCAGCCGCCTTCTTCAATA-3′


### Statistical Analyses

Statistical analyses were performed with SPSS 20.0 (IBM Statistics, Ehingen, Germany). HERV expression data were analyzed with bivariate correlation analyses (Spearman rank correlation) and Chi^2^ tests. Survival analyses were performed with Kaplan–Meier analyses and multivariate Cox’s Regressions analyses adjusted for resection status, localization of the tumor, tumor entity, and tumor stage (inclusion).

## Results

### HERV-K and -F Expression in Soft Tissue Sarcoma Samples

**FIGURE 1:**
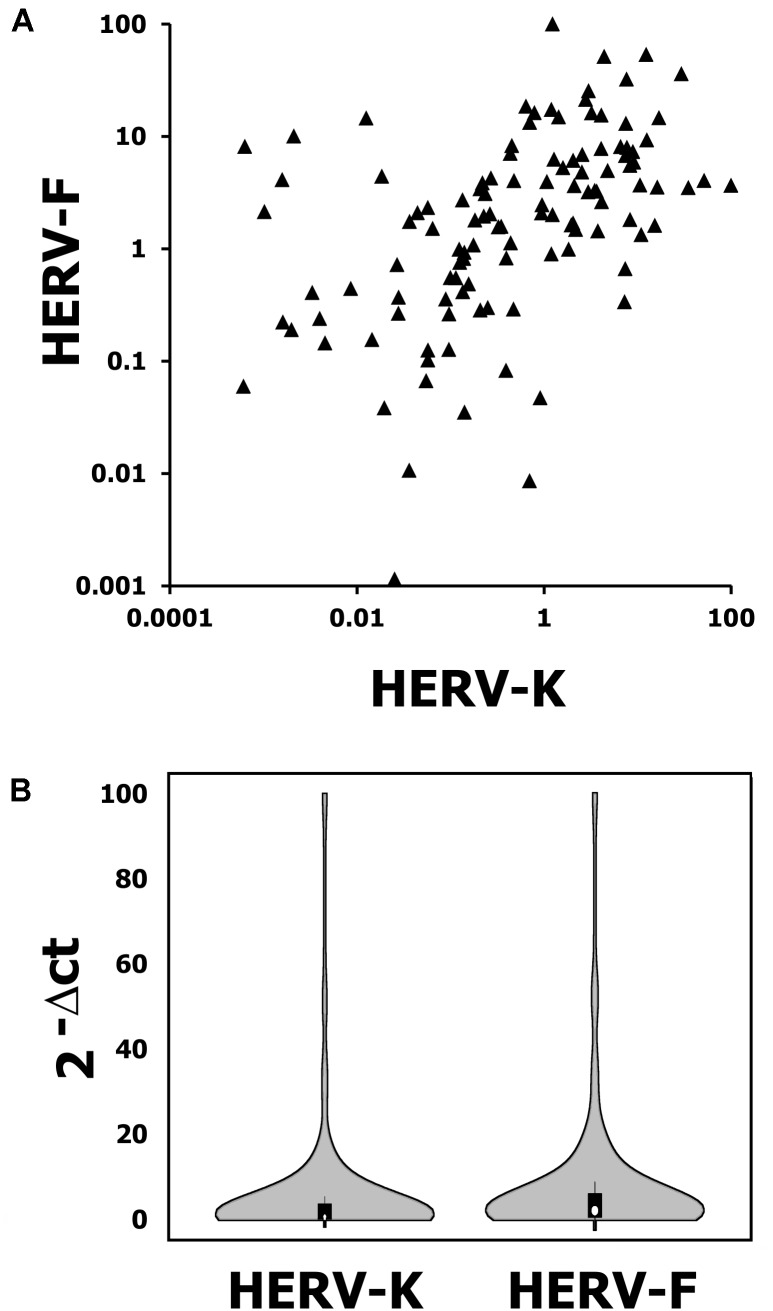
K vs. F blot **(A)** and violin blot **(B)** exhibiting the distribution of HERV-K and HERV-F mRNA expression in 120 analyzed soft tissue sarcoma (STS) specimens. Violin plot was generated with BoxPlotR (http://shiny. chemgrid.org/boxplotr/).

HERV-K mRNA expression was detected in 120 patients samples with a mean expression of 4.1 (range: 0.06–95.6; 2^-ΔCT^ value). HERV-F mRNA expression was also detected in 120 patients sarcoma specimen with a mean expression of 6.0 (range: 1 × 10^-5^–99.3; 2^-ΔCT^ value, see **Figures 1A,B**). Additionally, we measured the HERV-K and HERV-F mRNA expression in normal skeletal muscle tissue. HERV-K mRNA expression was determined at 0.093 (2^-ΔCT^ value) and HERV-F mRNA expression at 0.296 (2^-ΔCT^ value). Skeletal muscle tissue therefore exhibited lower HERV mRNA values than 76.6 and 78.3% of STS samples, respectively. For survival analyses, HERV-K or HERV-F mRNA expression were classified according to the median as cut-off value (HERV-K: 0.56; HERV-F: 2.02). In Chi^2^ tests, low or elevated HERV-K expressions exhibited no correlation to demographic (age, sex) or clinical parameters (tumor entity or localization, resection type, tumor size, number of relapses, and patients status). In contrast, a significant correlation of HERV-F expression with the histological subtype of the STS was observed (*p* = 0.047).

### Association of HERV Expression with Clinicopathological Parameters

In bivariate regression analyses, the association between HERV-K or HERV-F expression and several clinicopathological parameters were tested (see **Table 3**). Interestingly, HERV-F and HERV-K expression was significantly associated (*r*_S_ = 0.499; *p* = 6.4 × 10^-9^). Both HERV-F and HERV-K expression exhibited a significant inverse association with the actual tumor diameter at surgery (*r*_S_ = -0.309; *p* = 0.001 and *r*_S_ = -0.467; *p* = 7.4 × 10^-8^; respectively). Moreover, HERV-K mRNA expression was significantly inversely associated with BCL2 mRNA expression (*r*_S_ = -0.408; *p* = 0.0002) and miR-199a (*r*_S_ = 0.361; *p* = 0.0004) expression, while solely HERV-F expression was significantly associated with miR-203 expression (*r*_S_ = 0.333; *p* = 0.005). Intriguingly, both HERV-K and HERV-F expression were significantly associated with levels of hypoxia-related genes like HIF-1α mRNA expression (*r*_S_ = 0.444; *p* = 3.0 × 10^-6^ and 0.359; *p* = 0.0002; respectively) or miR-210 expression (*r*_S_ = 0.399; *p* = 0.001 and *r*_S_ = 0.366; *p* = 0.002; respectively). Additionally, both HERV-K and HERV-F expression were significantly associated to H2A.Bbd mRNA expression (*r*_S_ = 0.456; *p* = 0.0009 and *r*_S_ = 0.302; *p* = 0.012, respectively).

**Table 3:** Bivariate correlations (Spearman’s rank test; r_s_) of HERV-K or HERV-F mRNA expression with several clinicopathological and molecular parameters.

		*r*_s_	*p*	*n*
HERV-K mRNA	Tumor diameter	-0.309	**0.001**	120
	BCL2 mRNA expression	-0.408	**0.0002**	77
	miR-210 expression	0.399	**0.001**	71
	miR-203 expression	0.199	0.098	70
	HIF-1a mRNA expression	0.444	**3.0 × 10^-6^**	102
	miR-199a expression	0.361	**0.0004**	94
	H2A.Bbd expression	0.456	**0.0009**	68
	HERV-F mRNA expression	0.499	**6.4 × 10^-9^**	120
HERV-F mRNA	Tumor diameter	-0.467	**7.4 × 10^-8^**	120
	BCL2 mRNA expression	-0.207	0.071	77
	miR-210 expression	0.366	**0.002**	71
	miR-203 expression	0.333	**0.005**	70
	HIF-1a mRNA expression	0.359	**0.0002**	102
	miR-199a expression	0.116	0.264	94
	H2A.Bbd expression	0.302	**0.012**	68
	HERV-K mRNA expression	0.499	**6.4 × 10^-9^**	120


*Significant p-values are marked in bold.*

### HERV-K or -F Expression and Patients’ Survival

**FIGURE 2:**
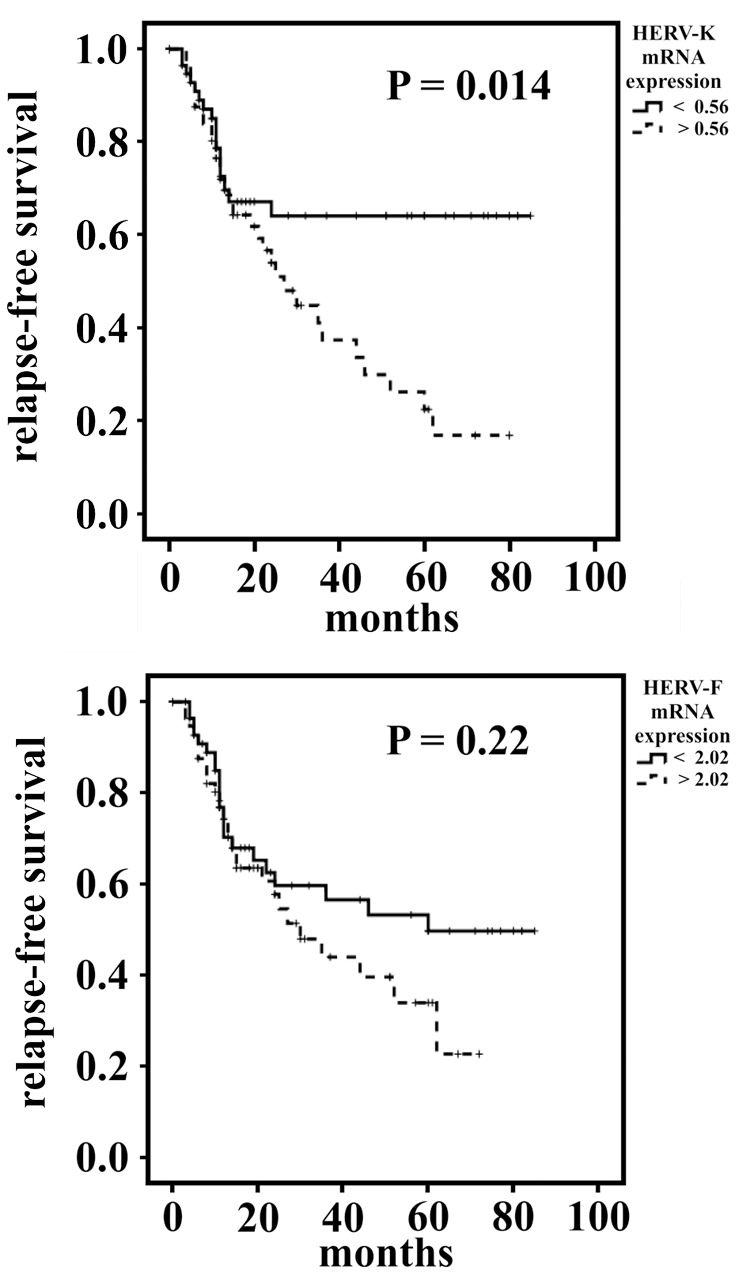
Survival analyses of STS patients with low or elevated intratumoral HERV-K **(A)** or HERV-F **(B)** expression. Kaplan–Meier survival blots for the relapse-free survival.

In Kaplan–Meier analyses, while HERV-F mRNA expression showed no significant correlation to patients’ disease-specific survival, a lower HERV-K mRNA expression was in trend associated with a worsened survival (*p* = 0.08; log rank test). Further, in a multivariate Cox’s regression analysis adjusted to the confounders resection type, tumor localization, tumor histotype and staging, there was no significant correlation between HERV-K or HERV-F mRNA expression and patient survival (see Supplementary Figure S6). Interestingly, when analyzing the relapse-free survival in Kaplan–Meier analyses, patients with a lower HERV-K mRNA expression exhibited a significantly longer relapse-free survival (*p* = 0.014; log-rank test, see **Figure 2A**). A comparable effect was observed in patients with lower HERV-F mRNA expression; however, there it was not significant (*p* = 0.22, see **Figure 2B**). Additionally, when analyzing the effect of the HERV-K mRNA expression on the relapse-free survival in a multivariate Cox’s regression analysis, an elevated HERV-K mRNA expression was in trend associated with a 1.78-fold increased risk for a relapse (*p* = 0.08). Furthermore, when comparing only patients exhibiting low HERV-F and HERV-K (*n* = 43) with patients exhibiting both elevated HERV-F and HERV-K expression (*n* = 43) in a multivariate Cox’s regression analysis, an elevated expression of HERVs was in trend significantly associated with a 2.08-fold increased relative risk for a relapse (*p* = 0.066).

## Discussion

In this study, we demonstrated that a robust mRNA expression of HERV-K and HERV-F in a cohort of 120 STS samples is detectable, and that the expression of HERV-K and HERV-F is correlated with clinicopathological features and hypoxia-related gene expression. Furthermore, an elevated HERV-K mRNA expression was significantly associated with a shorter relapse-free survival.

There are only few data on the expression of HERVs in STS. Schiavetti and colleagues studied the expression of HERV-K-MEL in sarcoma in comparison to the expression in a patient’s sample of melanoma cells. This transcript was detectable in 9/23 (39.1%) of sarcoma samples (Schiavetti et al., 2002). There is no data on the expression of HERV-F in sarcoma, however, one report shows a wide expression of HERV-F in tumor cell lines originating from mamma carcinoma, ovarian carcinoma, pancreatic adenocarcinoma, prostate carcinoma, glioblastoma, and others (Yi and Kim, 2004). Interestingly, HERV-F expression was not detected in any somatic tissue tested, with the exception of placenta (Yi and Kim, 2004). These reports are consistent with the assumption, that HERV sequences are normally methylated and therefore transcriptionally inactive, but are hypomethylated and activated during carcinogenesis (Kreimer et al., 2013; Hurst and Magiorkinis, 2017). Concordantly, the treatment with 5′-azacytdidine, a known DNA methyltransferase inhibitor, activates HERV sequences in diverse tumor cell lines (Strissel et al., 2012; Laska et al., 2013; Chiappinelli et al., 2015). Therefore, we propose that re-induction of HERVs may also occur during the multi-step process of sarcomagenesis.

Intriguingly, we identified a significant association of the HERV-K and -F expression with those of the hypoxia-related genes HIF-1α and miR-210. HIF-1α is a key regulator of the hypoxic response, and miR-210 is the most prominent microRNA upregulated by hypoxia (Kulshreshtha et al., 2007). There is little knowledge about interactions between HERV expression and hypoxic response. It has been described, that the hypoxia-mimetic CoCl_2_ increases the expression of ERV3 in Hodgkin’s lymphoma cell lines, and that this increase in ERV3 expression might be associated with a pro-apoptotic reaction (Kewitz and Staege, 2013). Other groups demonstrated upregulation of the HERV-W expression in neuroblastoma cell lines due to hypoxic conditions (Hu et al., 2016) or upregulation of the HERV-E expression in renal cell carcinomas due to inactivation of the von Hippel-Lindau factor and subsequent stabilization of the oxygen sensor protein HIF-1α (Cherkasova et al., 2011). In our *ex vivo* samples, we detected an inverse association between HERV-K and BCL2 mRNA expression, implying that HERV-K overexpression could be associated with apoptosis-induction. However, other reports performed on *in vitro* cell cultures demonstrate that the HERV-K family exerts an anti-apoptotic role (Broecker et al., 2016). Further research on this contradiction is warranted.

Furthermore, HERV-K and HERV-F expression were both significantly correlated to the mRNA expression of H2A.Bbd, a histone A2 variant encoded on the X chromosome, which is found to be associated with the nucleosomes of transcriptionally active genomic regions (Chadwick and Willard, 2001). H2A.Bbd was further shown to induce a more relaxed structure of the DNA by destabilizing the nucleosome (Bao et al., 2004; Doyen et al., 2006), which resembles the genomic reorganization induced by histone acetylation in a modification-independent manner (Eirín-López et al., 2008). A recent report showed H2A.Bbd to localize temporarily on replication-active DNA regions. By this mechanism, H2A.Bbd is holding the DNA in a more decondensed state, thereby increasing S-phase progression (Sansoni et al., 2014). Thus, it can be speculated that an H2A.Bbd overexpression in patient samples may be associated with a more transcriptionally active genome resulting in an increased chance of reactivation and expression HERV species.

In our patient cohort, we detected a significant association between a lower HERV-K expression and a longer relapse-free survival. This is concordant with previous reports describing a better overall prognosis for patients with a lower HERV-K expression in breast cancer (Golan et al., 2008; Zhao et al., 2011) or hepatocellular carcinoma (Ma et al., 2016). Additionally, hypomethylation and subsequent HERV induction was also demonstrated in ovarian carcinoma, and specifically the extent HERV-K hypomethylation was associated with a poor prognosis and therapy resistance in ovarian carcinoma patients (Menendez et al., 2004; Iramaneerat et al., 2011).

## Conclusion

To the best of our knowledge we present the first report suggesting an involvement of the HERV-K expression in the clinical course of STS. From our *ex vivo* data, we also suggest that HERV-K and -F expression may be regulated directly or indirectly by tumor hypoxia. Furthermore, HERV-K was associated to apoptosis in our samples, therefore may be an interesting therapeutic target.

## Author Contributions

MG performed the data analysis and revised the manuscript; SB, LO, and MK carried out the clinical sample processing and the qPCR measurements; PW recruited the patients and collected the tissue specimen; TG, HT, and MS conceived the study design, prepared and revised the manuscript. All authors read and approved the final version of the manuscript.

## Conflict of Interest Statement

The authors declare that the research was conducted in the absence of any commercial or financial relationships that could be construed as a potential conflict of interest.

## Footnotes

1 www.repeatmasker.org/
