# Supplementary material for: Elevated HERV-K Expression in Soft Tissue Sarcoma Is Associated with Worsened Relapse-Free Survival
Source: Front Microbiol. 2018 Feb 13;9:211. doi: 10.3389/fmicb.2018.00211 (PMC5816752; doi:10.3389/fmicb.2018.00211)
Supplement: Supplementary file 1 [file Presentation_1.PDF]

Supplementary Material

Elevated HERV-K expression in soft tissue sarcoma is associated with worsened relapse-free survival

M Giebler, MS Staege, S Blauschmidt, LI Ohm, M Kraus, P Würl, H Taubert, T Greither*

*** Correspondence:** Corresponding Author: thomas.greither@medizin.uni-halle.de

A

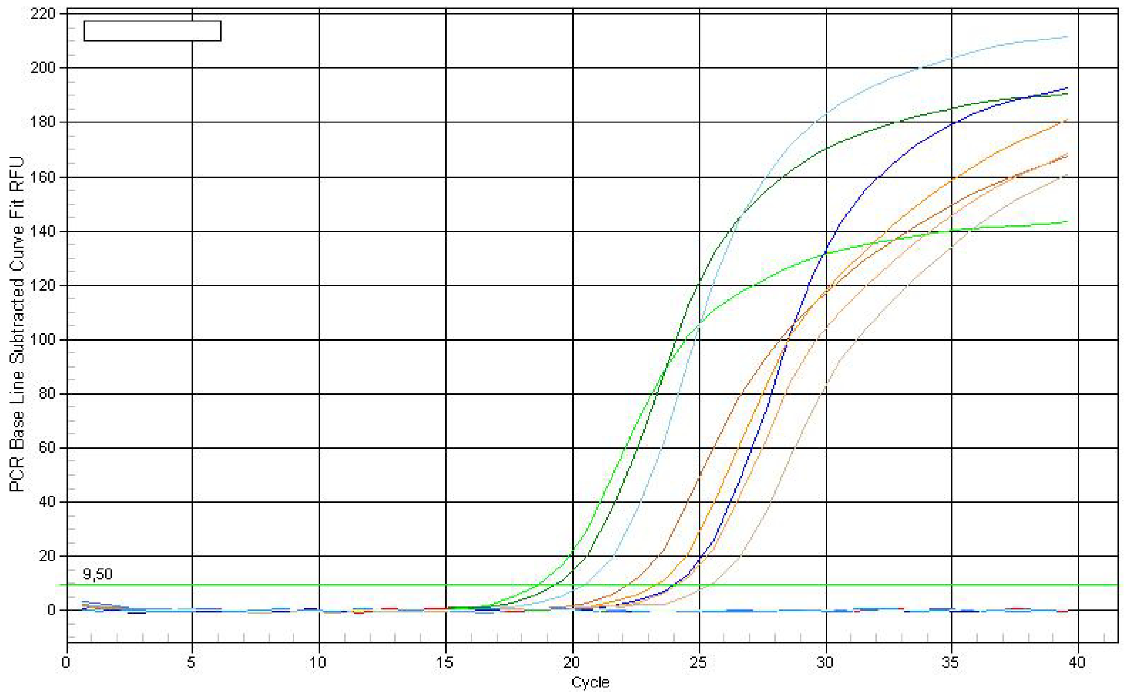

B

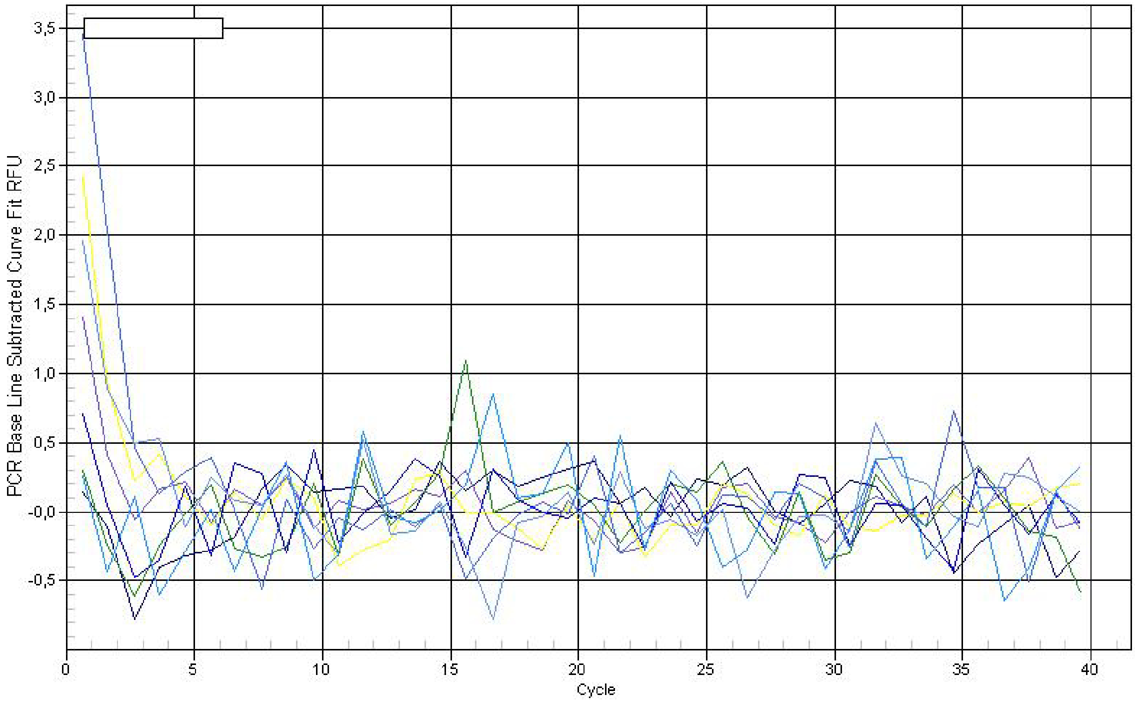

Supplementary Figure 1. Mock-RT experiments for HERV mRNA detection after cDNA synthesis reaction with or without reverse transcriptase. Melt curve analyses for all HERV-K and HERV-F qPCR reactions **(A)** or for qPCR reactions containing only the RT-negative samples **(B)**.

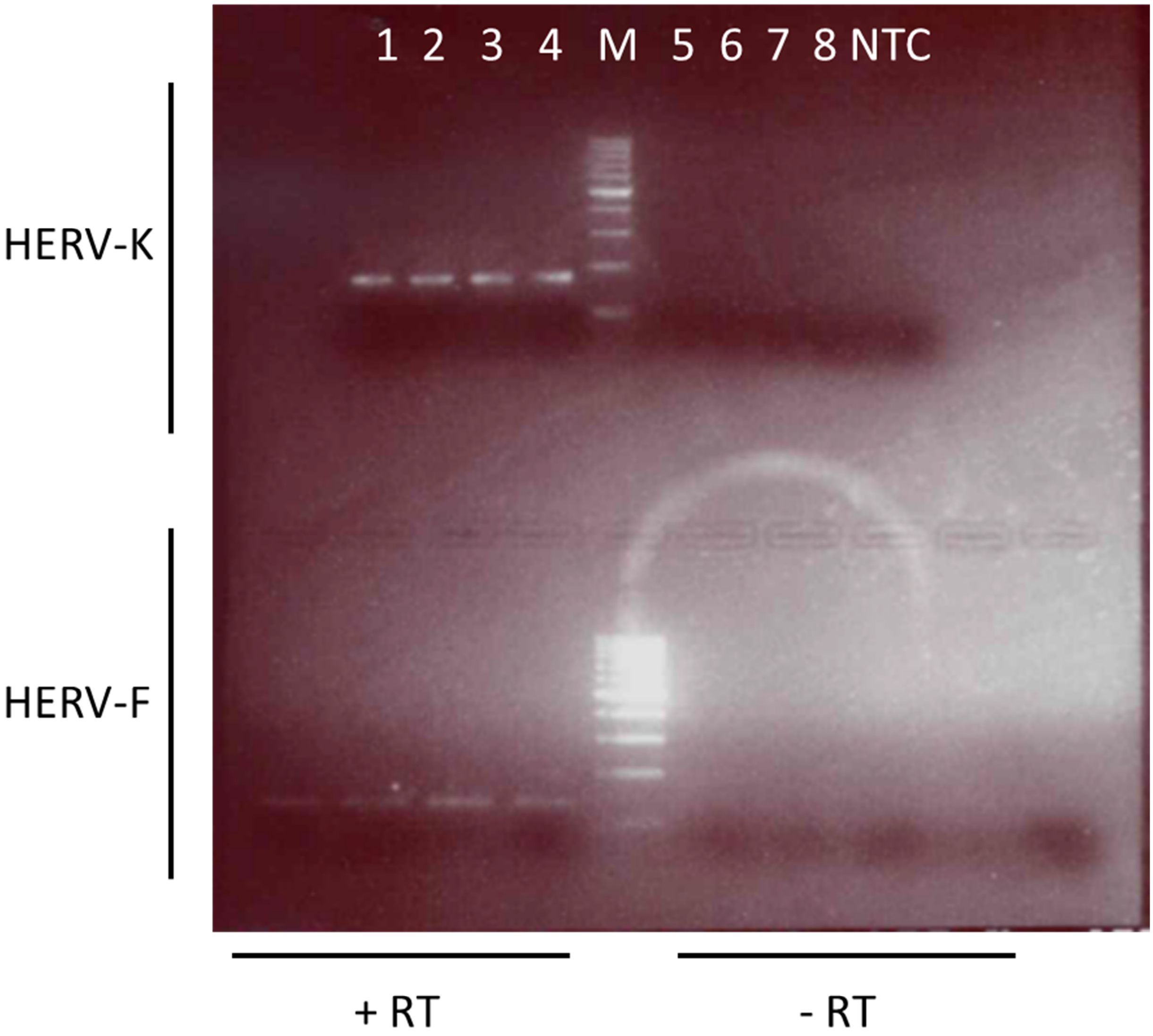

Supplementary Figure 2. Gel electrophoresis analysis of the mock-RT reactions. qPCR amplicons of cDNA synthesis reactions with or without reverse transcriptase after 40 PCR cycles. Legend: 1 – 4: soft tissue sarcoma samples, RT-positive; 5 – 8: same soft tissue sarcoma samples, RT-negative; NTC: non template control.

A

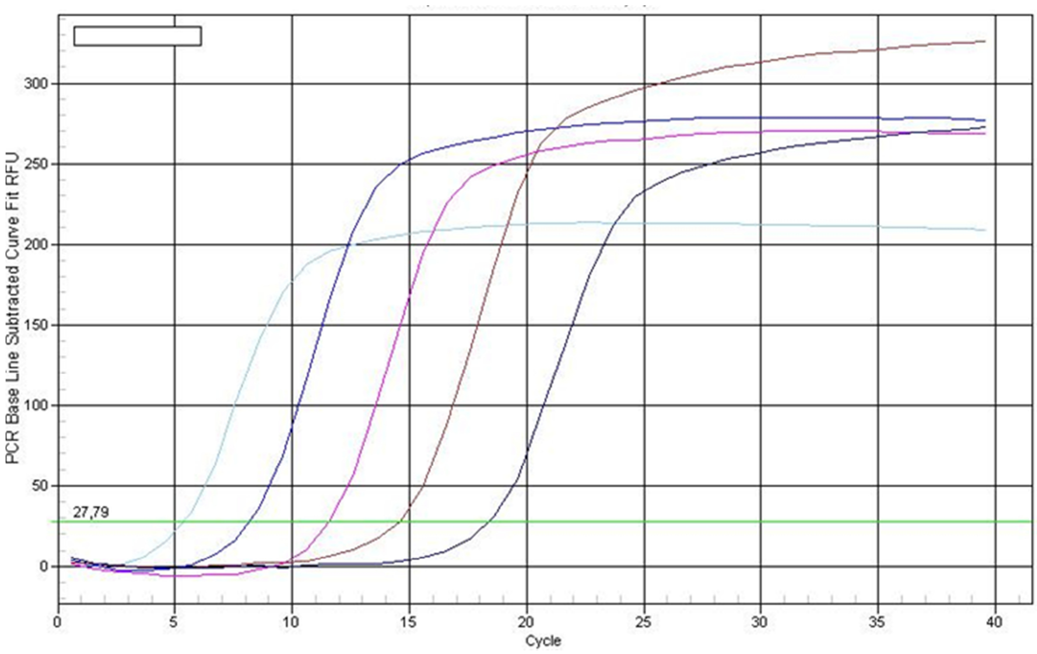

B

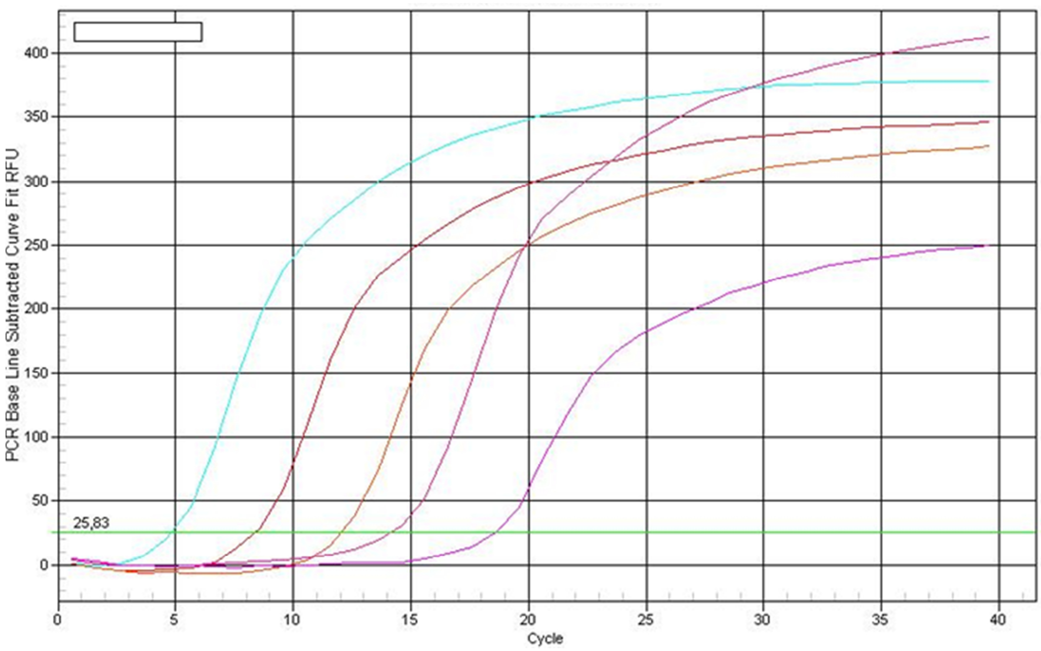

Supplementary Figure 3. qPCR analyses for the linearity of the PCR reaction of the HERV-K **(A)** and HERV-F **(B)** reaction. 1:10 – 1:100,000 dilutions of gel-extracted HERV-K and HERV-F amplificates were used for the PCR reaction.

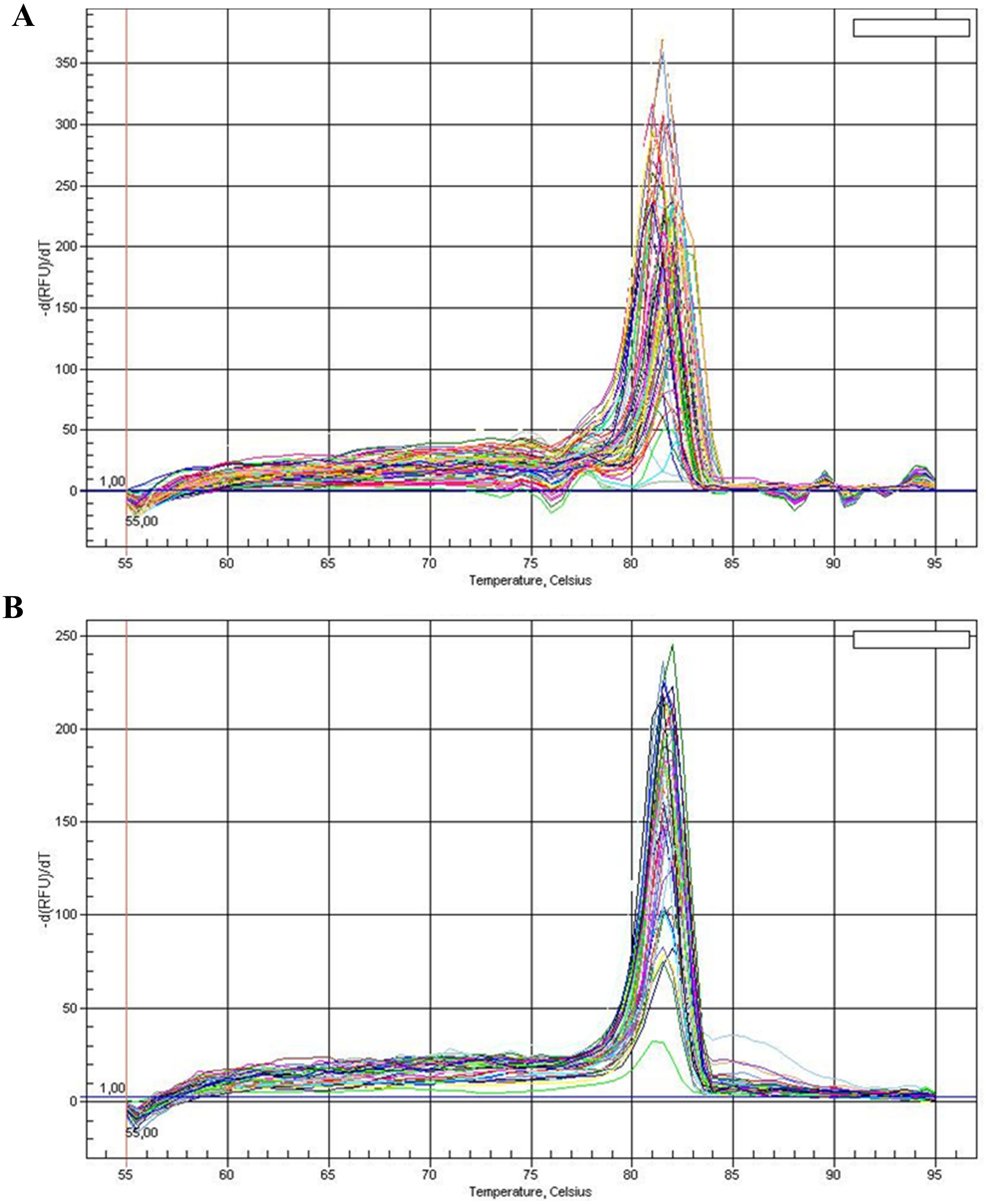

Supplementary Figure 4. Melt analyses of the HERV-K qPCR reaction for 75 **(A)** or 49 **(B)** soft tissue sarcoma patient samples analyzed. 120 of these samples showed single peaks. Four samples with multiple peaks (not included in the figures) were excluded from further analyses.

A

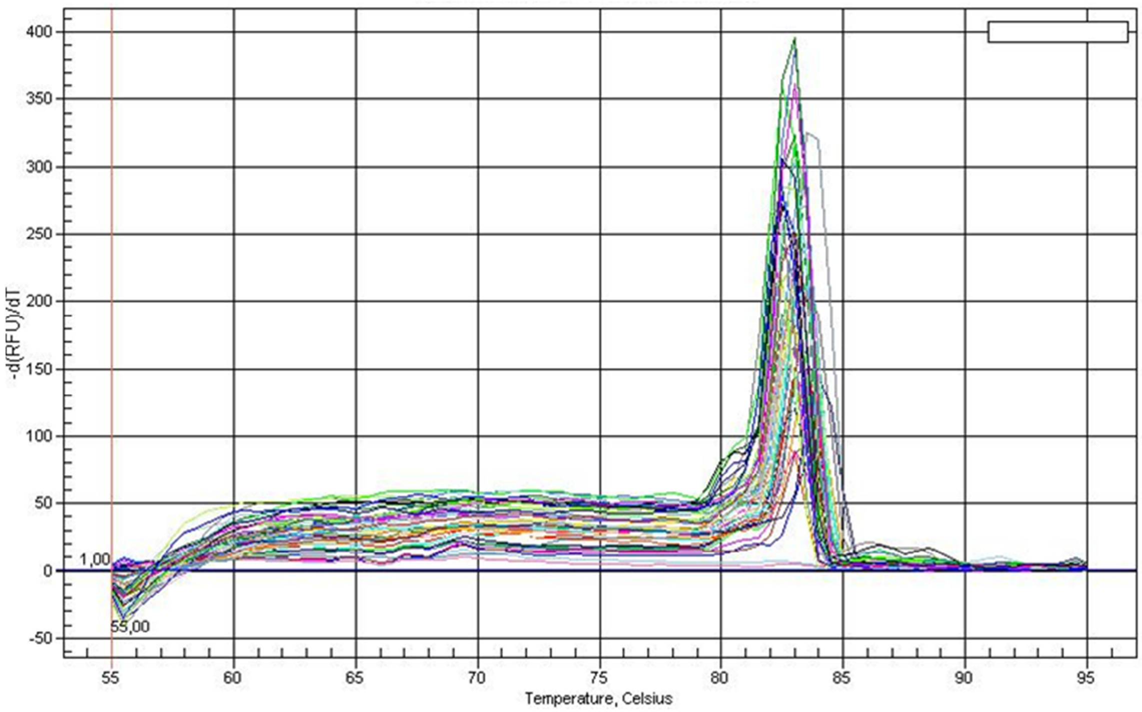

B

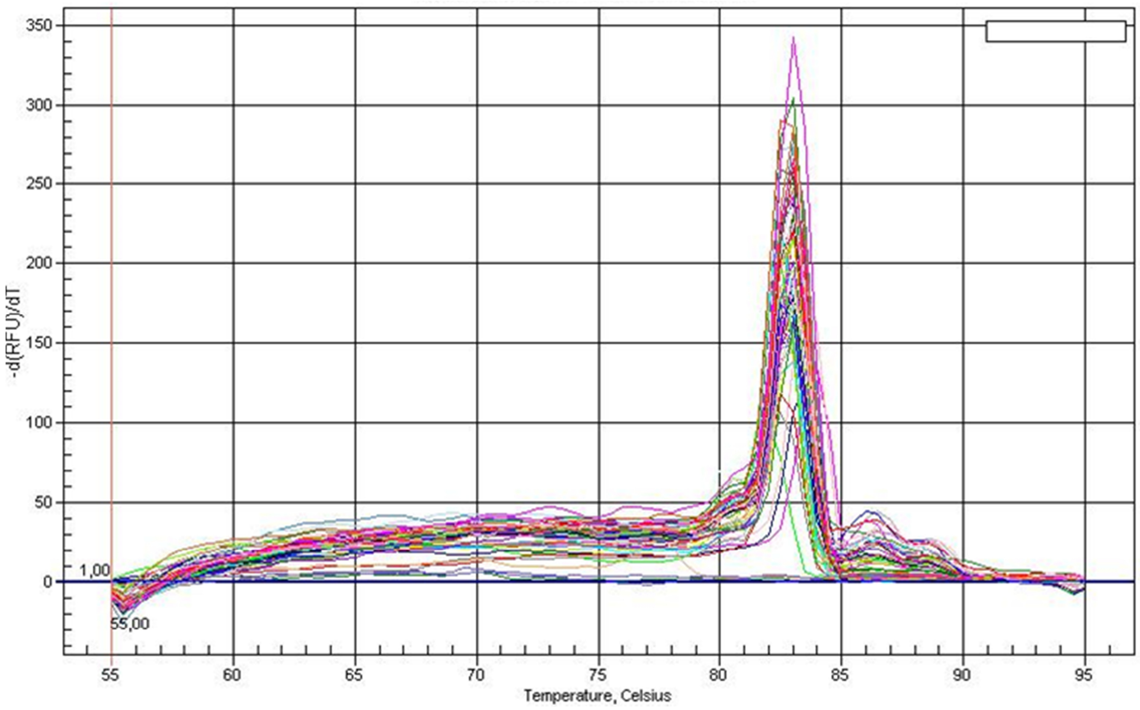

Supplementary Figure 5. Melt analyses of the HERV-F qPCR reaction for 75 **(A)** or 49 **(B)** soft tissue sarcoma patient samples analyzed. . 120 of these samples showed single peaks. Four samples with multiple peaks (not included in the figures) were excluded from further analyses.

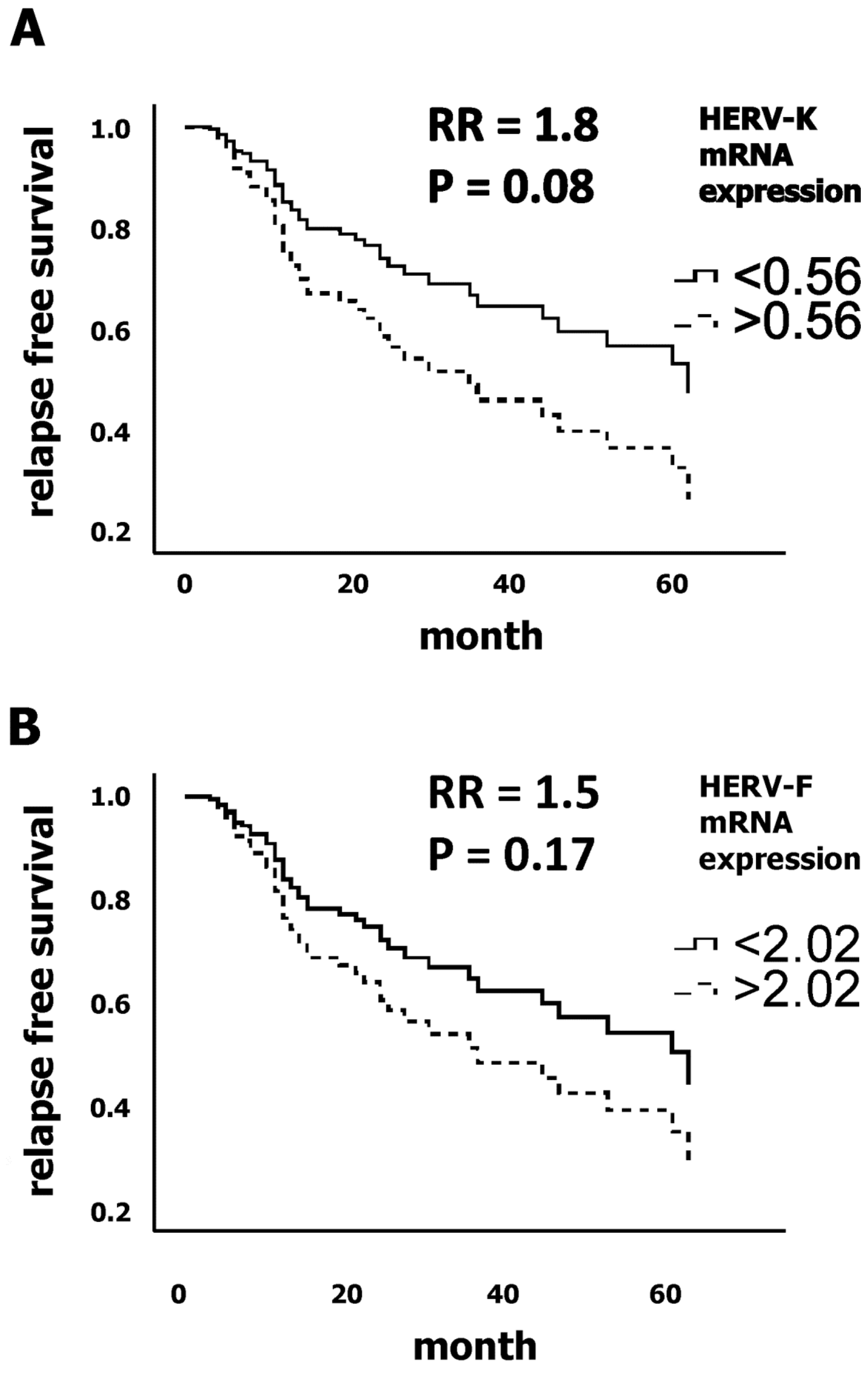

Supplementary Figure 6. Cox's regression analyses. HERV-K mRNA expression **(A)** and HERV-F mRNA expression **(B)** were analyzed regarding the relapse free survival of 120 soft tissue sarcoma patients. Abbreviation: RR = relative risk
